# Non-small cell lung cancer (NSCLC), EGFR downstream pathway activation and TKI targeted therapies sensitivity: Effect of the plasma membrane-associated NEU3

**DOI:** 10.1371/journal.pone.0187289

**Published:** 2017-10-31

**Authors:** Matilde Forcella, Monica Oldani, Samantha Epistolio, Stefania Freguia, Eugenio Monti, Paola Fusi, Milo Frattini

**Affiliations:** 1 Department of Biotechnologies and Biosciences, University of Milano-Bicocca, Milano, Italy; 2 Laboratory of Molecular Pathology, Institute of Pathology, Locarno, Switzerland; 3 Department of Molecular and Translational Medicine, University of Brescia, Brescia, Italy; Universita degli Studi di Parma, ITALY

## Abstract

Adenocarcinoma of Non-Small Cell Lung Cancer (NSCLC) is a severe disease. Patients carrying EGFR mutations may benefit from EGFR targeted therapies (e.g.: gefitinib). Recently, it has been shown that sialidase NEU3 directly interacts and regulates EGFR. In this work, we investigate the effect of sialidase NEU3 overexpression on EGFR pathways activation and EGFR targeted therapies sensitivity, in a series of lung cancer cell lines. NEU3 overexpression, forced after transfection, does not affect NSCLC cell viability. We demonstrate that NEU3 overexpression stimulates the ERK pathway but this activation is completely abolished by gefitinib treatment. The Akt pathway is also hyper-activated upon NEU3 overexpression, but gefitinib is able only to decrease, and not to abolish, such activation. These findings indicate that NEU3 can act directly on the ERK pathway through EGFR and both directly and indirectly with respect to EGFR on the Akt pathway. Furthermore, we provide evidence that a healthy mucosa cell line (with EGFR wild-type gene sequence) is slightly sensitive to gefitinib, especially in the presence of NEU3 overexpression, thus hypothesizing that NEU3 overexpressing patients may benefit from EGFR targeted therapies also in absence of EGFR point mutations. Overall, the expression of NEU3 may be a novel diagnostic marker in NSCLC because, by its ability to stimulate EGFR downstream pathways with direct and indirect mechanisms, it may help in the identification of patients who can profit from EGFR targeted therapies in absence of EGFR activating mutations or from new combinations of EGFR and Akt inhibitors.

## Introduction

Lung cancer is the leading cause of cancer death in both sexes [1]; it is generally classified in Small Cell Lung Cancer (SCLC) and Non-Small Cell Lung Cancer (NSCLC), the latter accounting for approximately 85–95% of all lung cancers. Among NSCLC, adenocarcinomas (AC) are the most frequent histotype, representing 40% of diagnosed patients.

Current conventional treatment for lung cancer consists of surgery for operable patients, followed by chemo/radiotherapy. However, the prognosis is usually poor especially for patients with advanced disease. In this setting, the introduction of targeted therapies has led to improved outcome for AC patients; one such target is the epidermal growth factor receptor (EGFR), which is frequently overexpressed and aberrantly activated in NSCLC [2]. When EGFR binds to several specific ligands, multiple signalling pathways are activated including the RAS/RAF/ERK/MAPK pathway, resulting in cell proliferation, and the PI3K/Akt pathway, STAT (Signal Transducers and Activators of Transcription) 3 and 5 signal transduction pathways, resulting in the evasion of apoptosis [3]. EGFR has been exploited as a molecular target of two different kinds of molecules: monoclonal antibodies (mAbs), directed against the extracellular domain and interfering with receptor dimerization (like Cetuximab and Panitumumab) and tyrosine kinase inhibitors (TKI), blocking the intracellular receptor kinase activity [4]. mAbs against EGFR are active when EGFR is altered through protein expression, typically occurring in colorectal (CRC) cancer, while TKIs can inhibit the EGFR protein when a mutation occurs in its tyrosine kinase, encoded by exons 18–21. The latter is the typical EGFR activation found in lung cancer patients, occurring in 10–40% of patients, more frequently in Asians, females, non-smokers, and in adenocarcinomas. Over the last decade, a variety of TKI have received Food and Drug Administration (FDA) approval for treating NSCLC, among which Gefitinib (Iressa) and Erlotinib (Tarceva) are currently in use for advanced and metastatic NSCLC in the first line of treatment [5–7].

However, not all EGFR mutations in the tyrosine kinase domain display the same effect with respect to TKI efficacy: in-frame deletions in exon 19 as well as L858R and L861Q point mutations in exon 21 are associated with the best response to TKI. Point mutations occurring in exon 18 (in codons 709 and 719) are associated with an intermediate response, while alterations in exon 20 lead to TKI resistance. One of the last mutations, the T790M change, is the typical mechanism of acquired resistance occurring in patients treated with gefitinib or erlotinib: therefore, patients developing such a mutation must be treated with another type of TKI (i.e.: irreversible TKI, or second-generation TKI)[8–11].

Sialidases (EC 3.2.1.18), or neuraminidases, are widely distributed glycohydrolases, removing sialic acid residues from a variety of glycoconjugate [12]. In humans, four sialidases with different subcellular localizations and biochemical features have been described: a lysosomal sialidase (NEU1), a cytosolic sialidase (NEU2), a plasma membrane-associated sialidase (NEU3) and a mitochondrial/endoplasmic reticulum (ER) sialidase (NEU4) [12]. Defects in glycosylation are known to play a role in cancer malignancy [13], being associated with invasiveness and metastatic potential in cancer cells [14].

Among sialidases, the plasma membrane-associated NEU3 [15] is involved in the regulation of many trans-membrane signalling processes [16,17] and has been shown to act not only on gangliosides within its own membrane, but also on gangliosides belonging to the plasma membranes of neighbouring cells [18]. Moreover, several studies have shown that NEU3 is up regulated in most cancers, including melanoma, colon, renal, ovarian and prostate cancers NEU3 mRNA levels have been observed to increase 3- to 100-fold in human colon cancer tissues compared with adjacent non-tumour mucosal tissues [19]. More recently, a link between NEU3 and EGFR activation pathway has been demonstrated. Following the initial finding that human sialidase NEU3 co-immunoprecipitates with EGFR in HeLa cells [20], it has been found that NEU3 is directly involved in EGFR desialylation in colorectal cancer, promoting receptor dimerization, and therefore EGFR activation [21]. Given the importance of EGFR activation in NSCLC pathogenesis, we investigated the effect of sialidase NEU3 overexpression on EGFR downstream pathway activation and TKI targeted therapies sensitivity in a series of lung cancer cell lines.

## Materials and methods

### Cell cultures

The human non-small cell lung cancer (NSCLC) cell lines HCC4006 (ATCC® CRL-2871™), H1650 (ATCC® CRL-5883™), H2228 (ATCC® CRL-5935™), HCC78 (DSMZ ACC 563), H1975 (ATCC® CRL-5908™), H1734 (ATCC® CRL-5891™) were grown in RPMI 1640 medium supplemented with heat-inactivated 10% foetal bovine serum (FBS), 2 mM L-glutamine, 100 U/ml penicillin and 100 μg/ml streptomycin. The normal human lung cell line HSAEC1 (ATCC® CRL-4050™) was grown in SABM Basal Medium™ supplemented with Bovine μPituitary Extract (BPE), Hydrocortisone, human Epidermal Growth Factor (hEGF), Epinephrine, Transferrin, Insulin, Retinoic Acid, Triiodothyronine, Bovine Serum Albumin–Fatty Acid Free (BSA-FAF), 100 U/ml penicillin and 100 μg/ml streptomycin. All cell lines were maintained at 37°C in a humidified 5% CO_2_ incubator.

The American Type Culture Collection (Rockville, MD, USA) validated cell lines by short tandem repeat profiles that are generated by simultaneous amplification of multiple short tandem repeat loci and amelogenin (for gender identification).

All the reagents for cell culture were supplied by Lonza (Lonza Group, Basel, Switzerland).

### RNA isolation and qPCR

Cells were seeded at 5 × 10^5^ cells/60 mm dish and after 24 h the total RNA was isolated from cells using RNeasy Mini Kits (Qiagen, Chatsworth, CA, USA), according to manufacturer’s instructions. RNA was reverse-transcribed using SuperScript® II RT (Invitrogen, Carlsbad, CA, USA), oligo dT and random primers, according to the manufacturer’s protocol.

For quantitative real-time PCR (qPCR), SYBR Green method was used to evaluate NEU3 expression. Briefly, 50 ng cDNA was amplified with SYBR Green PCR Master Mix (Applied Biosystems, Foster City, CA, USA) and specific primers (100 nM), using an initial denaturation step at 95°C for 10 min, followed by 40 cycles of 95°C for 15 sec and 59°C annealing for 1 min. Each sample was analysed for NEU3 expression and normalized for total RNA content using Pol2 gene as an internal reference control. The relative expression level was calculated with the Livak method (2^[-ΔΔC(T)]^) [22] and was expressed as a fold change ± standard deviation. The accuracy was monitored by the analysis of melting curves.

The following primers were used: NEU3 Fw 5’-TGAGGATTGGGCAGTTGG-3’ and Rv 5’-CCCGCACACAGATGAAGAA-3’; EGFR Fw 5’-GGTGTGTGCAGATCGCAAAG-3’ and Rv 5’-GACATGCTGCGGTGTTTTCAC-3’; Pol2 Fw 5’-AGGAGCAAAGCCTGGTGTT-3’ and Rv 5’-ACCCAAAGCTGCCAGAAGT-3’.

### Vector

cDNA coding for human sialidase NEU3 was previously subcloned into plasmid pcDNA3.1 (Invitrogen, Carlsbad, CA, USA), in frame with C-terminal hemagglutinin (HA) epitope [23].

### Transfection

Cells were seeded at 5 × 10^5^ cells/60 mm dish, 1.5 × 10^6^ cells/100 mm dish or at 1 × 10^4^ cells/well into a 96-well plates and, after 24 h, transiently transfected either with pcDNA3.1 vector containing wild-type NEU3 cDNA or with the empty vector as a control (mock) in a 10% serum medium using JetPEI^TM^ DNA transfection reagent (Polyplus transfection SA, France), according to the manufacturer’s instructions. After transfection, cells were grown in a complete medium.

### Membrane fractionation

HSAEC1, HCC4006 and H1734 cell lines were seeded at 1.5 × 10^6^ cells/100 mm dish and after 24 h transiently transfected either with pcDNA3.1 vector containing wild-type NEU3 cDNA or with the empty vector. After 36 h *post*-transfection cells were rinsed with ice-cold PBS and harvested by scraping in PBS containing protease inhibitors. Cells were broken by bundle sonication on ice and after cell lysis samples were clarified by centrifugation at 800 g for 10 min at 4°C. The resulting supernatant (total cell extract) was centrifuged at 100 000 g for 1 h at 4°C in order to obtain a total cell membrane fraction. The resulting pellet was resuspended in PBS containing protease inhibitors and analysed in Western-blot for NEU3 detection.

### SDS-PAGE and Western-blot

To examine the effect of EGF, sialidase NEU3 and gefitinib on the phosphorylation of EGFR and EGFR downstream members, the human non-small cell lung cancer cell lines HCC4006 and H1734 and the normal human lung cell line HSAEC1 were seeded at 5 × 10^5^ cells/60 mm dish, transiently transfected and 36 h after transfection pre-treated with 1 μM gefitinib for 3 h before exposure to 20 ng/mL EGF for 15 min at 37°C. Gefitinib was provided by Cell Signaling Technology (Danvers, MA, USA). EGF was purchased by Sigma (St. Louis, MO, USA).

The cells were then rinsed with ice-cold PBS and lysed in RIPA buffer, containing protease and phosphatase inhibitors and 1 mM PMSF. After lysis on ice, homogenates were obtained by passing 5 times through a blunt 20-gauge needle fitted to a syringe and then centrifuged at 15,000 g for 30 min. Supernatants were analysed for protein content by the BCA protein assay [6].

SDS-PAGE and Western-blot were carried out by standard procedures. 60 μg of proteins were separated on 10% acrylamide/bis-acrylamide SDS-PAGE, transferred onto a nitrocellulose membrane (Millipore, Billerica, MA, USA), probed with the appropriated antibodies and visualized using ECL detection system (Millipore, Billerica, MA, USA). Protein levels were quantified by densitometry of immunoblots using Scion Image software (Scion Corp., Frederick, MD, USA). The following primary antibodies (all purchased by Cell Signaling Technology, Danvers, MA, USA) were used: anti EGFR (dilution 1:1000), phospho-EGFR (Tyr1068; dilution 1:1000), p44/42 MAPK (ERK 1/2; dilution 1:1000), phospho-p44/42 MAPK (ERK 1/2) (Thr202/Tyr204; dilution 1:1000), Akt (dilution 1:1000), phospho-Akt (Ser473; dilution 1:1000), PTEN (dilution 1:1000), vinculin (dilution 1:10000) and GAPDH (dilution 1:10000). For the analysis of the membrane samples the following primary antibodies were used: anti NEU3 (dilution 1:250) purchased by Thermo Fisher Scientific (Waltham, MA USA) and anti-transferrin receptor (dilution 1:500) purchased by Invitrogen (Carlsbad, CA, USA).

IgG HRP-conjugated secondary antibodies (purchased by Cell Signaling Technology, Danvers, MA, USA) were diluted 1:10000.

### Cell viability through MTT assay

In order to evaluate gefitinib sensitivity, cells were seeded at a density of 1 × 10^4^ cells/well into a 96-well plates and after 24 h were treated with various gefitinib concentrations (0, 0.01, 0.1, 1, 10 μM).

After 72 h at 37°C, the medium was replaced with a complete medium without phenol red and 10 μl of 5 mg/mL MTT [3-(4,5-dimethylthiazol-2)-2,5-diphenyltetrazolium bromide] solution (Sigma, St. Louis, MO, USA) were added to each well. After a further 4 h incubation time, absorbance upon formed formazan crystals solubilisation with 10% Triton-X-100 in acidic isopropanol (0.1 N HCl) was measured at 570 nm using a micro plate reader. Viabilities were expressed as a percentage of the untreated controls. The 50% growth inhibition (IC_50_) was determined from dose-response curve. Each experiment was performed in three replicate wells for each drug concentration and results are presented as the median of at least three independent experiments.

In order to assay the effect of NEU3 on cell viability in the presence of 27 nM or 1 μM gefitinib, cells were seeded in 96-well plates at a density of 1 × 10^4^ cells/well and after 24 h were transiently transfected. After 4 h from the addition of the jetPEI/DNA mixture to the cells, the medium was changed and the cells were treated with 27 nM or 1 μM gefitinib. After an incubation at 37°C for 36 h post transient transfection, the medium was replaced with complete medium without phenol red and 10 μl of 5 mg/ml MTT solution were added to each well. After a further 4 h incubation time, absorbance upon solubilisation was measured at 570 nm using a micro plate reader. Viabilities were expressed as a percentage of the mock.

### Propidium iodide staining of dead cells after gefitinib treatment

Cells were seeded at a density of 2.5 x 105 cells/well into a 12-well plates and after 24 h were treated with different gefitinib concentrations (0, 0.01, 0.1, 1, 10 μM). After 72 h at 37°C, the medium was recovered and the cells were trypsinized, harvested by centrifugation at 1200 rpm for 10 min and washed once with PBS. The supernatant was removed and the cells were resuspended in 500 μL of PBS containing 10 μL of 50 μg/mL propidium iodide solution. The suspension was mixed gently, incubated 1 min in the dark and analyzed on the flow cytometer equipped with an argon ion laser at 488 nm (Cytoflex S, Beckman Coulter, Brea, CA, USA), acquiring 10 000 events.

### Statistical analysis

Statistical analyses were performed by Student’s t-test. Three biological replicates were performed and samples were compared to their reference controls. Calculations were performed in R [24] using the heavy package [25] and multcomp package [26]. The significance was defined as p<0.05.

## Results

### Sensitivity to gefitinib of different lung cell lines

**Fig 1:**
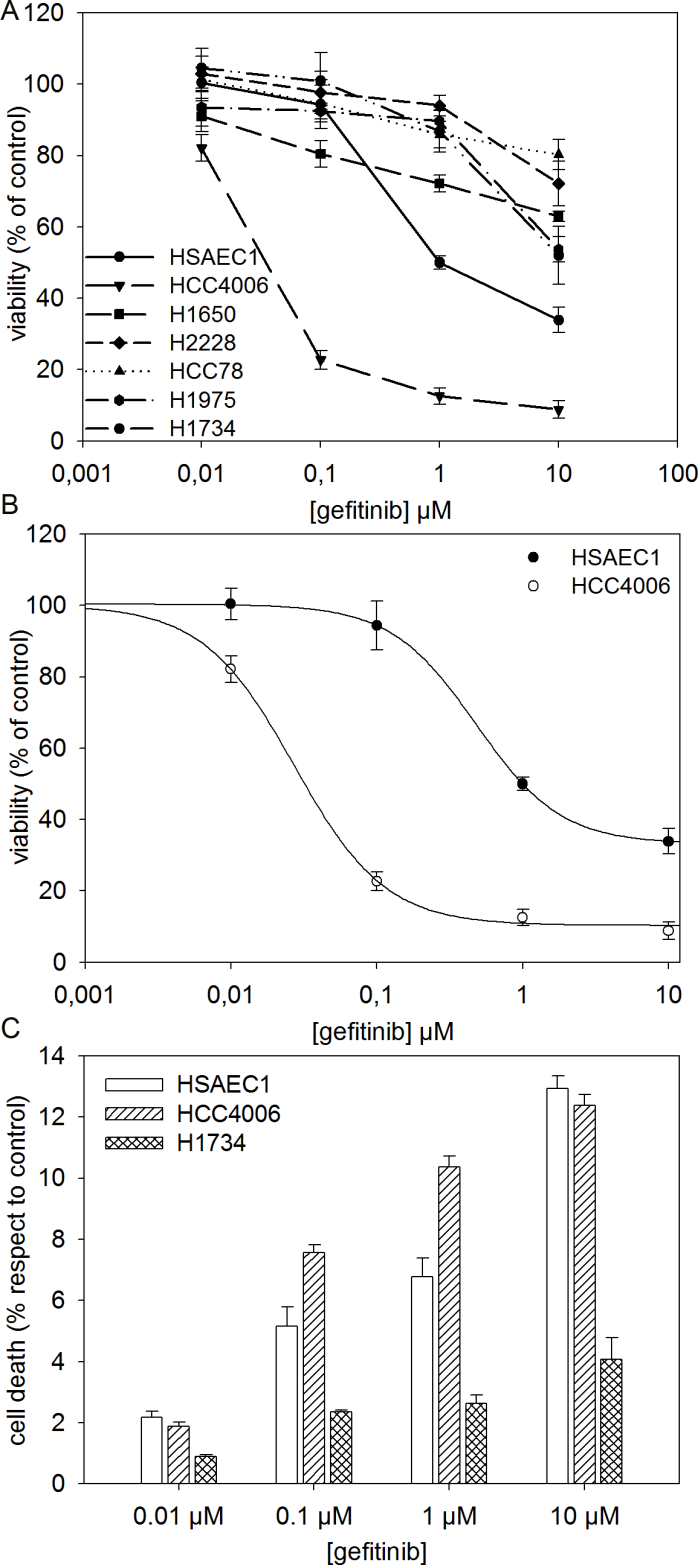
Sensitivity to gefitinib. (A) Dose-response curves of human lung cell lines to gefitinib. Cell survival was determined by MTT assay in the absence or the presence of different gefitinib doses (0, 0.01, 0.1, 1 and 10 μM) for 72 h. Viabilities are expressed as a percentage of the untreated control ± standard error (SE). (B) Nonlinear Regression of experimental data for HSAEC1 and HCC4006 cells lines was obtained using a Four Parameter Logistic Curve f1 = min + (max-min)/(1 + (x/EC_50_)^(-Hillslope)). (C) Cell death of HSAEC1, HCC4006 and H1734 cells lines was determined by flow cytometry through PI staining. The cells were treated with different gefitinib concentration ranging from 0.01 to 10 μM for 72 h and stained by PI dye. The zero concentration was defined as a control and cell death was expressed as a percentage of the control ± standard error (SE).

We first compared the effect of gefitinib on a panel of 6 NSCLC cell lines and one normal human lung cell line. Dose-response curves to gefitinib (0, 0.01, 0.1, 1 and 10 μM), assessed by MTT assay, are reported in Fig 1A. Five of the six NSCLC cell lines showed resistance to gefitinib (IC_50_ ⩾ 10 μM), four of which showed an EGFR wild-type sequence and an alteration in other relevant oncogenes. More in detail: H2228 cells, carrying an ALK translocation; HCC78 cells, characterized by a ROS1 rearrangement; H1975 cells, carrying a double EGFR mutation (the L858R change, an activating mutation and the T790M change, the best-known mechanism of EGFR-TKI primary and acquired resistance); and, finally, H1734 cells, presenting the KRAS G13C mutation. The fifth cell line resistant to gefitinib was H1650, which, although carrying an activating EGFR exon 19 deletion (del E746-A750), is unsensitive to TKI inhibitors through an unknown mechanism, as already reported in the literature [27]. On the contrary, HCC4006 cells, which are characterized by the classical ΔE746-A750 in EGFR exon 19, showed high drug sensitivity. The IC_50_ value of 27 ± 3 nM obtained for HCC4006 cells, by nonlinear regression of experimental data (Fig 1B), is reported in Table 1. Surprisingly, the normal human lung cell line HSAEC1 showed a partial sensitivity to gefitinib, with an IC_50_ value of to 478 ± 15 nM, showing an 18-fold lower sensitivity to gefitinib than HCC4006 cell line, but an at least 20-fold higher sensitivity than resistant non-small cell lung cancer cell lines.

**Table 1:** IC_50_ values for gefitinib.

Cell line	Gefitinib IC_50_ (nM, mean ± SE)
HSAEC1	478 ± 15
HCC4006	27 ± 3

IC_50_ was defined as the concentration that resulted in a 50% decrease of viability in MTT assay.

To evaluate cell death, cells were subjected to PI staining cytofluorimetric assay. Results are reported in Fig 1C: at 1 μM gefitinib, death rate corresponds to 7% and 10% for healthy HSAEC1 cells and for sensitive HCC4006 cells respectively. By comparing these data to cell viabilities reported in Fig 1A (about 50% and 12% for healthy HSAEC1 and sensitive HCC4006 cells, respectively) we are able to estimate the cytostatic effect of gefitinib, amounting to 43% and 78% for healthy HSAEC1 and sensitive HCC4006 cells, respectively. Moreover, PI staining confirmed that H1734 cells are resistant to gefitinib treatment, cell death amounting to 2% at 1 μM gefitinib, with an inhibition of proliferation of 11%.

### Sialidase NEU3 and EGFR are not overexpressed in lung cancer cell lines

**Fig 2:**
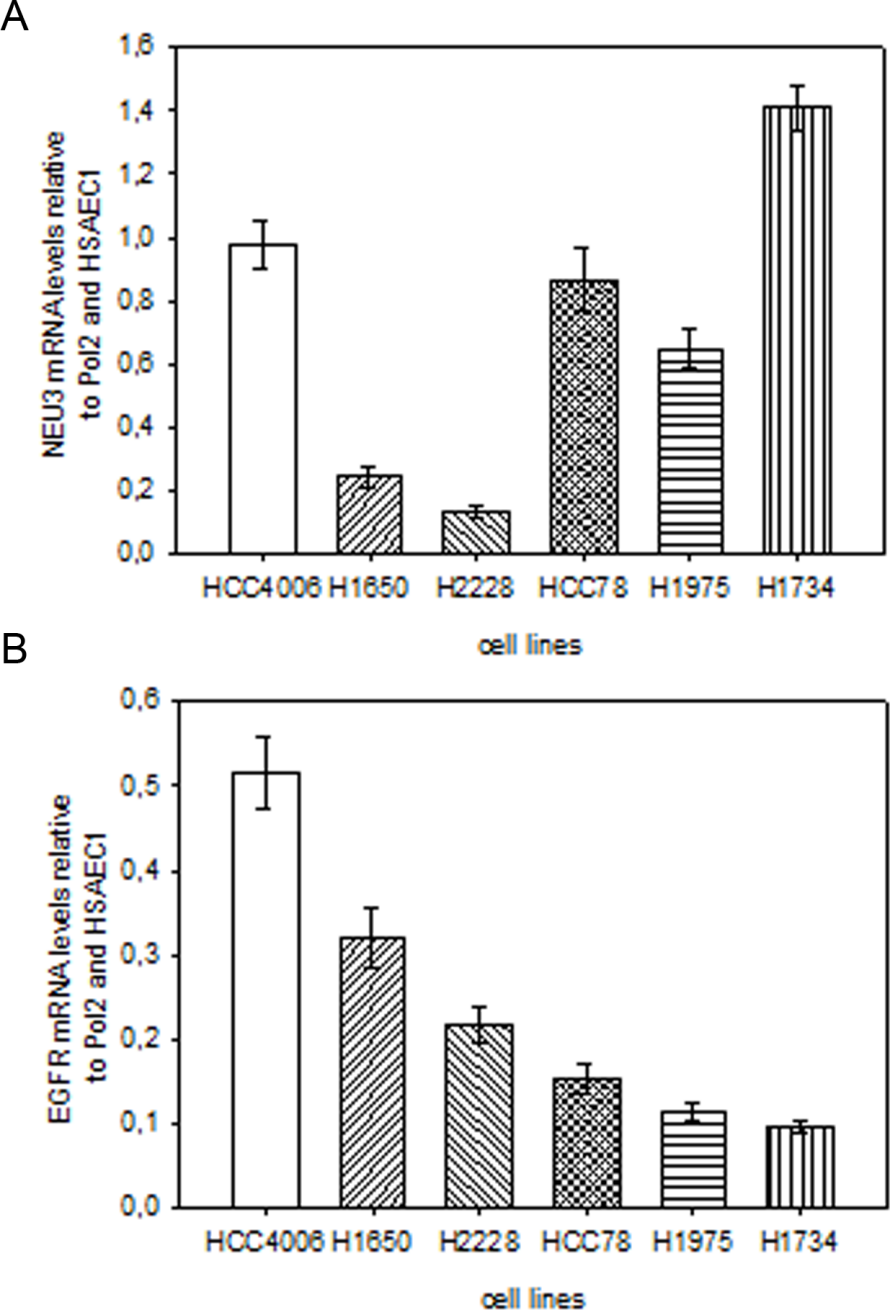
Relative quantification of *NEU3* and *EGFR* mRNA levels by quantitative real-time PCR in lung cancer cell lines. The relative expression levels of sialidase *NEU3* (A) and *EGFR* (B) in lung cancer cell lines were calculated with the Livak method (2^[-ΔΔC(T)]^) and were expressed as a fold change, using *Pol2* gene as internal reference control and the HSAEC1 normal lung cell line as calibrator. Values are presented as means ± standard error (SE).

We analyzed *NEU3 and EGFR* mRNA levels using qPCR, by comparing mRNA levels in lung cancer cells with those observed in the healthy HSAEC1 lung cell line. As shown in Fig 2, *NEU3* and *EGFR* were not overexpressed in any of the analyzed lung cancer cell lines; NEU3 mRNA levels were never significantly higher than 1, while EGFR mRNA levels were found downregulated in all cases, with a highest value of 0.5.

### NEU3 mRNA level and protein expression after transfection: membrane localization

**Fig 3:**
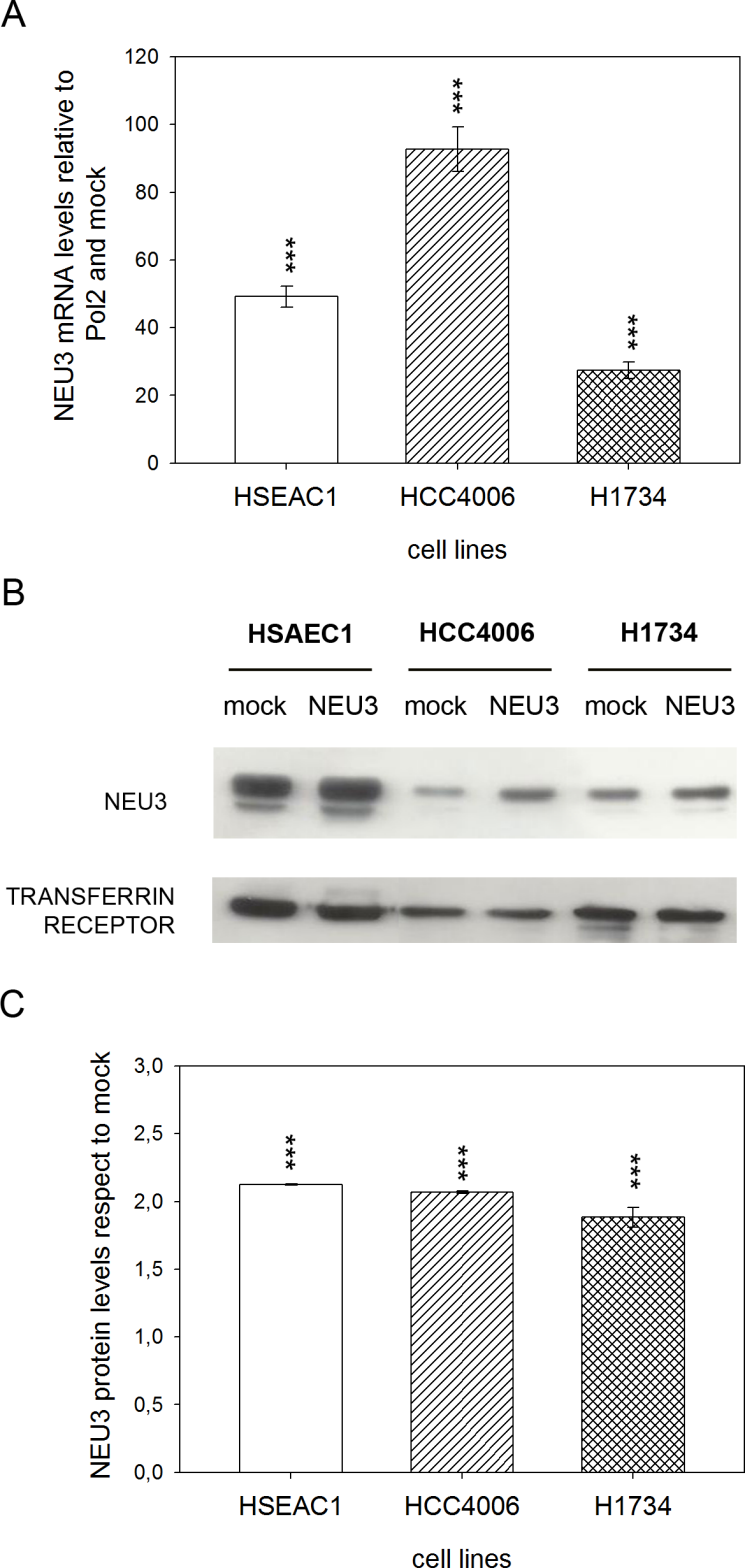
NEU3 transfection evaluation and NEU3 membrane localization. (A) Relative quantification of NEU3 mRNA levels by qPCR on HSAEC1, HCC4006 and H1734 cell lines transfected with either the empty vector (mock) or pcDNA3.1-HsNEU3. (B) Representative Western-blot analyses performed on membrane fractions. Proteins were separated on a 10% SDS-PAGE and probed with anti-NEU3 antibody. Transferrin receptor was used as a membrane marker. The experiments were performed in quadruplicate. (C) Densitometric analysis was performed with Scion Image Software. Values are expressed by comparing the data obtained after transfection with pcDNA3.1-HsNEU3 with those obtained after transfection with the empty vector (mock). Values are presented as means ± standard error (SE). ***p<0.001 (Student’s t-test).

We considered three of the cell lines previously analysed to perform NEU3 transfection experiments: the HCC4006 gefitinib sensitive cell line, the gefitinib resistant H1734 cell line and the HSAEC1 healthy lung cell line as a control. qPCR experiments, carried out after transfection with either the empty vector (mock) or pcDNA3.1-HsNEU3, revealed an increase in NEU3 mRNA levels of 50-fold for HSAEC1, 100-fold for HCC4006 and 30-fold for H1734 cell line compared to controls (Fig 3A). Cell membrane fractions were prepared after NEU3 transfection and Western-blot experiments confirmed its localization at the plasma membrane, according to the transferrin receptor presence, used as a membrane marker (Fig 3B). Densitometric analysis carried out comparing the data obtained after transfection with pcDNA3.1-HsNEU3 with those obtained after transfection with the empty vector (mock) revealed a FOLD-protein increase equal to 2 in all cases (Fig 3B).

### Human NEU3 sialidase overexpression does not affect cell viability

The effect of NEU3 transfection on cell viability was investigated, using MTT assay, in healthy lung cell line HSAEC1 and in lung cancer cell lines HCC4006 and H1734.

**Fig 4:**
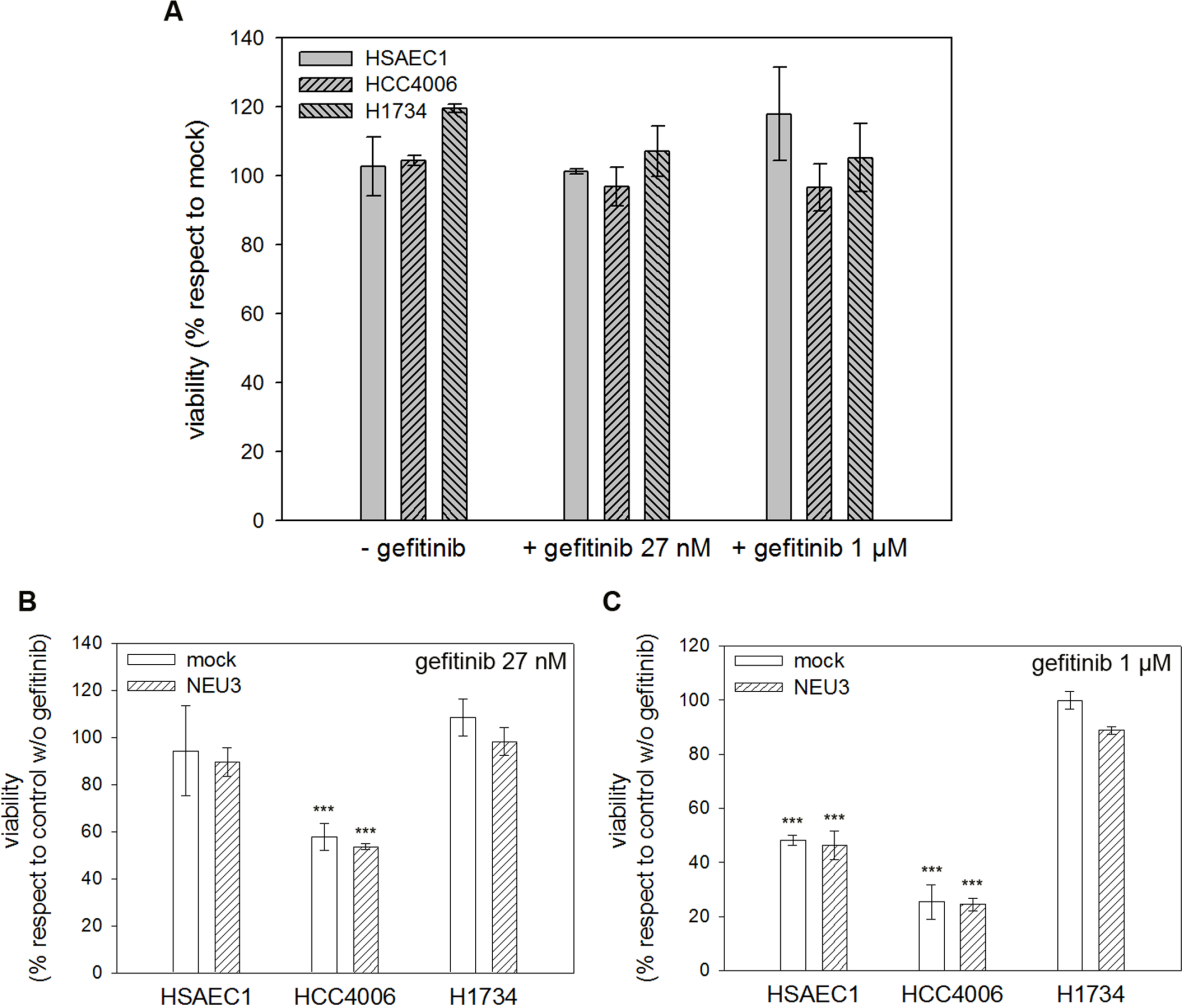
Evaluation of sialidase NEU3 effect on cell viability with or without gefitinib. MTT test was performed on HSAEC1, HCC4006 and H1734 cell lines transfected with either the empty vector (mock) or pcDNA3.1-HsNEU3 and then treated or not with 27 nM or 1 μM gefitinib for 36 h *post*-transfection. Data were normalized on control cells transfected with the empty vector (A). Cell viabilities of mock and NEU3 transfected cells are reported after treatment with either 27 nM (B) or 1 μM gefitinib (C). Data were normalized on control cells without drug. Values are presented as means ± standard error (SE).

Transfected cells were treated or not with either 27 nM (corresponding to HCC4006 IC_50_) or 1 μM gefitinib for 36 h *post*-transfection. Results showed no difference in cell viability following transfection with sialidase NEU3 in comparison to the mock, both in the absence and in the presence of gefitinib in all cell lines analyzed (Fig 4A). Viabilities of transfected and mock cells are shown separately in Fig 4B (at 27 nM gefitinib) and in Fig 4C (at 1 μM gefitinib). Results show that at 27 nM gefitinib sensitive HCC4006 cells viability is significantly reduced (57% for mock cells, 53% for transfected cells) and that at 1 μM gefitinib viability of both healthy HASAEC1 and sensitive HCC4006 cells is significantly reduced (48% and 46% for mock and transfected HSAEC1 cells, respectively; 25% and 24% for mock and transfected HCC4006 cells, respectively).

### Effect of EGF, NEU3 overexpression and gefitinib on EGFR, Akt and ERK phosphorylation

We examined the effect of EGF, NEU3 overexpression and gefitinib on the phosphorylation of EGFR and its downstream effectors, such as Akt and ERK, by Western-blot analysis in HSAEC1, HCC4006 and H1734 cell lines. Cells transfected either with empty vector (mock) or pcDNA3.1-HsNEU3, were treated or not with 1 μM gefitinib for 3 h and then stimulated or not with 20 ng/mL EGF for 15 min. We analyzed our data evaluating the effect of EGF in cells transfected or not with sialidase NEU3 and in the absence or the presence of gefitinib; the effect of NEU3 overexpression was evaluated in cells treated or not with either EGF or gefitinib, while the inhibitory effect of gefitinib was assayed in cells transfected or not with sialidase NEU3 and stimulated or not with EGF.

**Fig 5:**
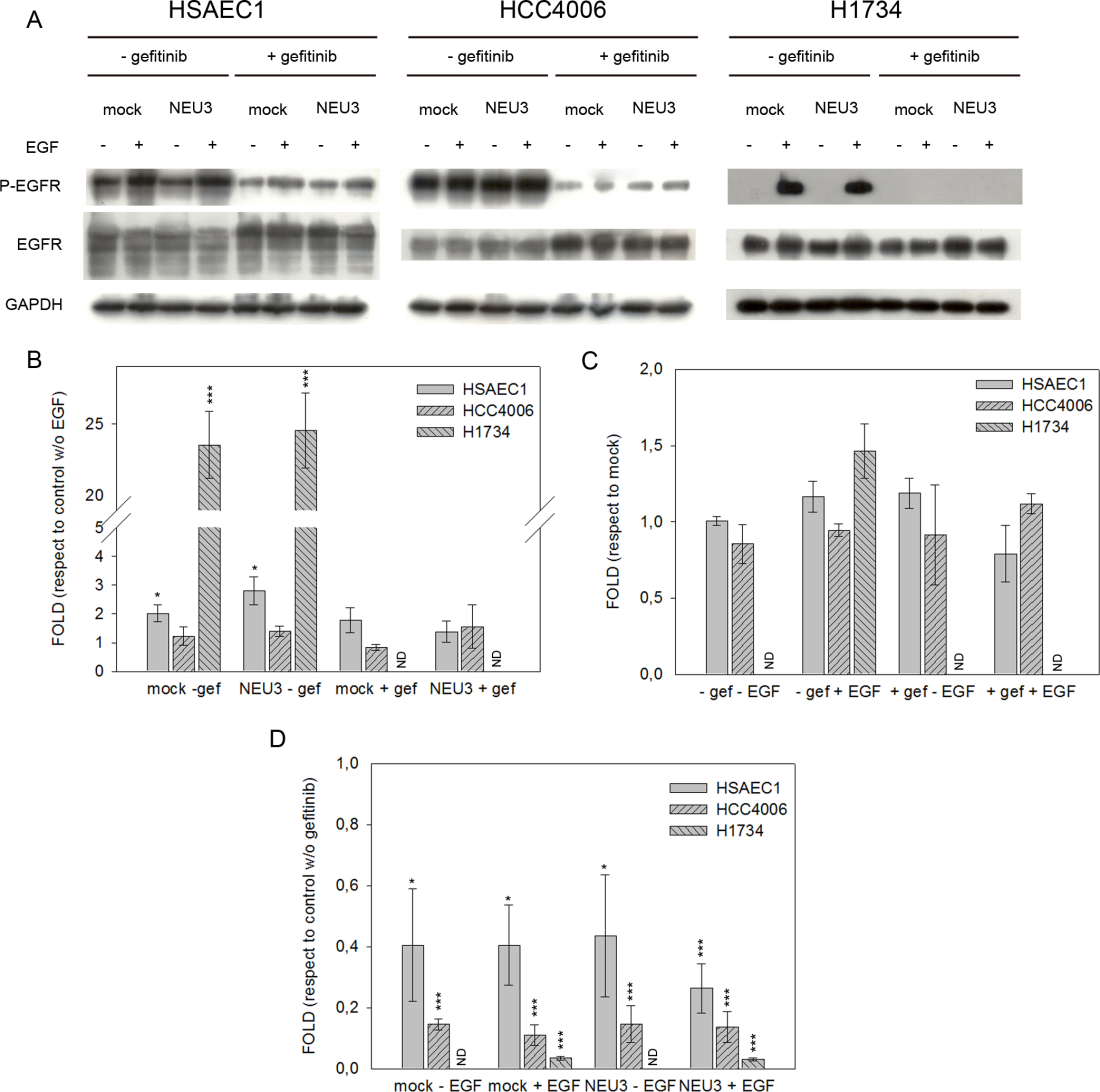
EGFR phosphorylation levels after EGF stimulation, sialidase NEU3 overexpression and gefitinib treatment. (A) Representative Western-blot analyses performed on a normal lung cell line (HSAEC1) and NSCLC cell lines (HCC4006 and H1734) transfected either with the empty vector (mock) or pcDNA3.1-HsNEU3. Cells were treated for 3 h with 1 μM gefitinib, followed by the addition of EGF (20 ng/mL) for 15 min. Protein extracts were separated on a 10% SDS-PAGE and probed with anti-EGFR, anti-P-EGFR antibodies. GAPDH was used as a loading control. The experiments were performed in triplicate. (B)–(C)–(D) Densitometric analysis was performed with Scion Image Software. Values are expressed by comparing the data obtained after EGF stimulation with those obtained in the absence of EGF (B); by comparing the data obtained after transfection with NEU3 with those obtained after transfection with the empty vector (mock) (C); by comparing the data obtained after gefitinib treatment with those obtained without gefitinib administration (D). Statistical analyses were performed using Student’s t-test. Values are presented as means ± standard error (SE). *p<0.05, **p<0.01, and ***p<0.001 (Student’s t-test).

Fig 5 shows EGFR phosphorylation levels in the different conditions. EGF administration stimulated EGFR autophosphorylation in healthy HSAEC1 cells (2- and 3-fold in mock cells and in NEU3 transfected cells, respectively) and in resistant H1734 cells (23-fold in both situations), in the absence of gefitinib and irrespective of NEU3 overexpression. Treatment with gefitinib abolished EGF effect in both cell lines (Fig 5A and 5B).

In the sensitive HCC4006 cells, we did not observe any EGF stimulation (Fig 5A and 5B), even in the absence of gefitinib, the cells showing a high basal level of phosphorylated EGFR; however, gefitinib administration lead to a decrease in EGFR phosphorylation.

In all the analyzed conditions, NEU3 overexpression did not lead to an increase in EGFR activation, as reported in Fig 5C.

The inhibitory effect of gefitinib on EGFR phosphorylation was observed in all cell lines, but to a different extent in each case (Fig 5A and 5D). In healthy HSAEC1, gefitinib caused a 60% inhibition compared to the mock cells, both in the absence and the presence of EGF. Upon NEU3 overexpression and treatment with EGF, HSAEC1 cells became more susceptible to inhibition by gefitinib (70%).

In HCC4006 cell line we observed a >80% gefitinib-induced inhibition of EGFR phosphorylation in all the situations. However, the highest inhibition (> 90%) was observed in H1734 cells overexpressing NEU3 upon EGF stimulation (Fig 5D). Furthermore, in H1734 drug-resistant cells no EGFR phosphorylation was detected in the absence of EGF stimulation.

**Fig 6:**
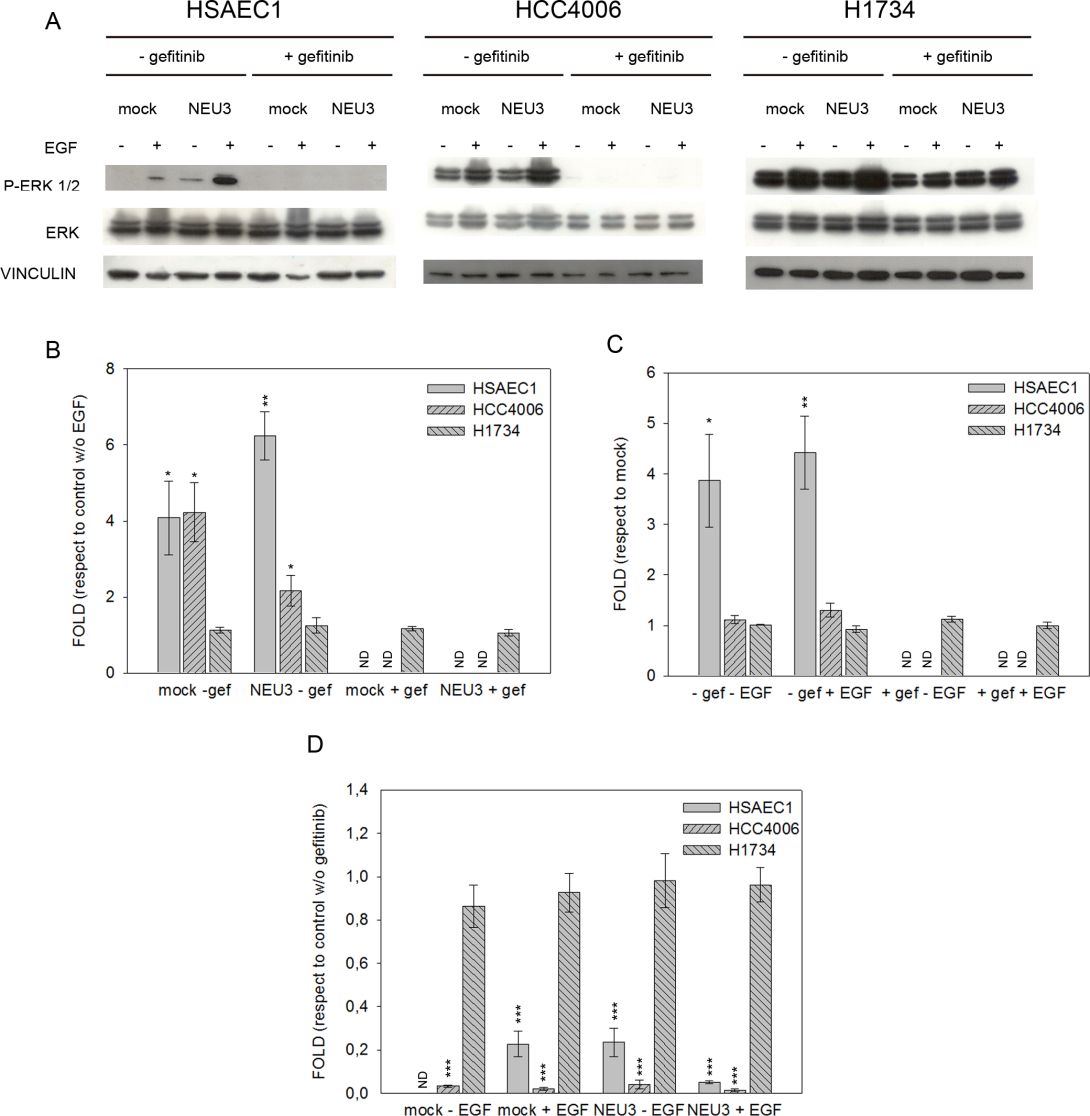
ERK phosphorylation levels after EGF stimulation, sialidase NEU3 overexpression and gefitinib treatment. (A) Representative Western-blot analyses performed on normal lung cell line (HSAEC1) and NSCLC cell lines (HCC4006 and H1734) transfected with either the empty vector (mock) or pcDNA3.1-HsNEU3. Cells were treated for 3 h with 1 μM gefitinib, followed by the addition of EGF (20 ng/mL) for 15 min. Protein extracts were separated on a 10% SDS-PAGE and probed with anti-ERK1/2, anti-P-ERK1/2 antibodies. Vinculin was used as a loading control. The experiments were performed in triplicate. (B)–(C)–(D) Densitometric analysis was performed with Scion Image Software. Values are expressed by comparing the data obtained after EGF stimulation with those obtained without EGF (B); by comparing the data obtained after transfection with NEU3 with those obtained after transfection with the empty vector (mock) (C); by comparing the data obtained after gefitinib treatment with those obtained without gefitinib administration (D). Statistical analyses were performed using Student’s t-test. Values are presented as means ± standard error (SE). *p<0.05, **p<0.01, and ***p<0.001 (Student’s t-test).

ERK phosphorylation levels, also evaluated under different conditions, are reported in Fig 6. We observed EGF stimulation of ERK phosphorylation in all cell lines, only in the absence of gefitinib (Fig 6A and 6B). In HSAEC1 and HCC4006 cells, ERK phosphorylation levels increased 4-fold after EGF administration, in the absence of NEU3 overexpression (mock). NEU3 overexpression, led to a 6-fold and a 2-fold increase in EGF stimulated ERK phosphorylation in HSAEC1 and HCC4006 lines, respectively. In H1734 cells we did not observe any significant increase of ERK phosphorylation level after EGF treatment (Fig 6A and 6B).

An effect of NEU3 overexpression on ERK phosphorylation, leading to a 4-fold increase, was observed only in healthy HSAEC1 lung cells, both treated and untreated with EGF, in the absence of gefitinib (Fig 6A and 6C).

ERK activation was completely inhibited by gefitinib both in HSAEC1 and HCC4006 cell lines, while gefitinib did not affect ERK phosphorylation level in the drug-resistant H1734 cell line (Fig 6A and 6D).

**Fig 7:**
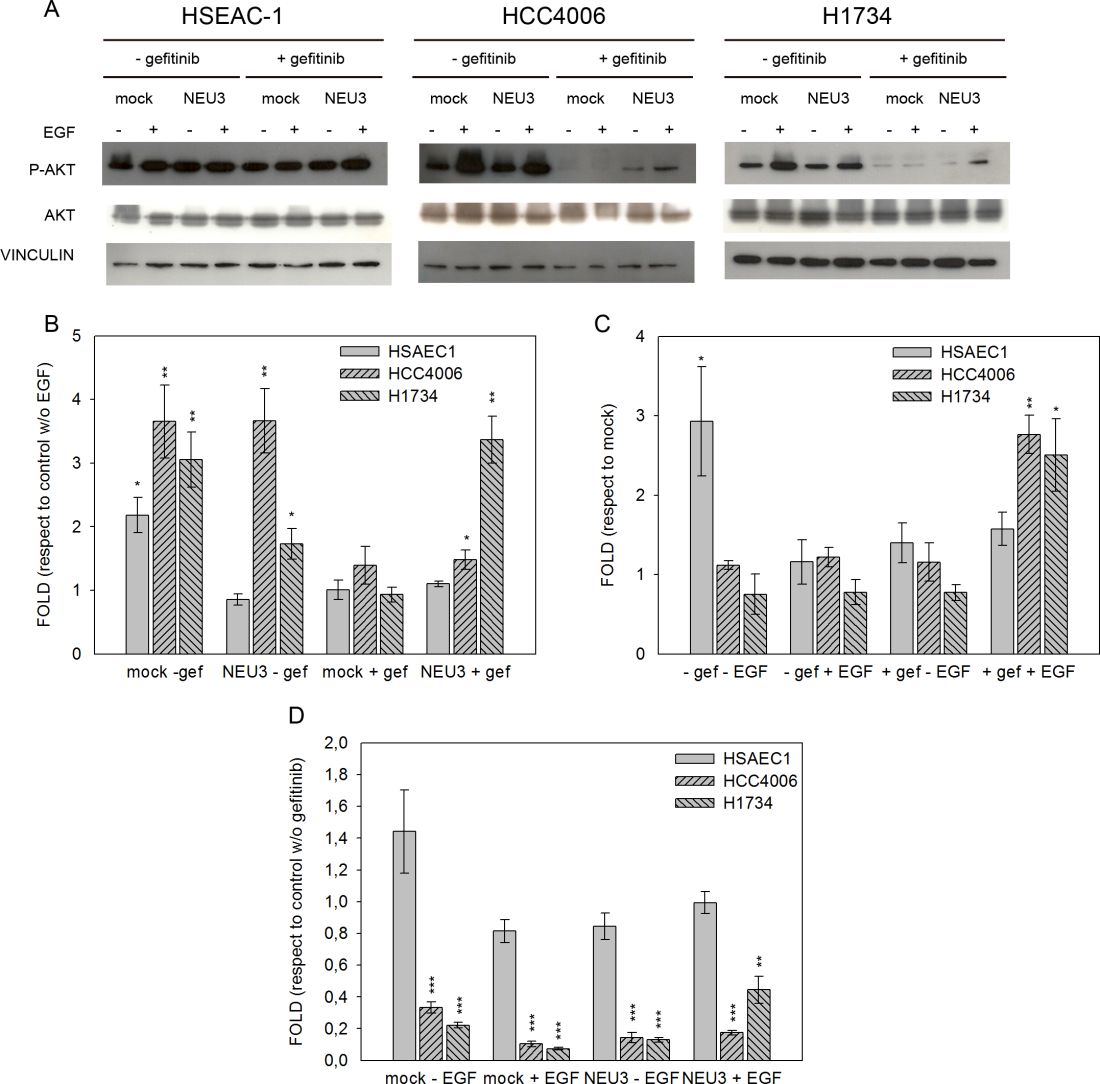
Akt phosphorylation levels after EGF stimulation, sialidase NEU3 overexpression and gefitinib treatment. (A) Representative Western-blot analyses performed on normal lung cell line (HSAEC1) and NSCLC cell lines (HCC4006 and H1734) transfected with either the empty vector (mock) or pcDNA3.1-HsNEU3. Cells were treated for 3 h with 1 μM gefitinib, followed by the addition of EGF (20 ng/mL) for 15 min. Protein extracts were separated on a 10% SDS-PAGE and probed with anti-Akt, anti-P-Akt antibodies. Vinculin was used as a loading control. The experiments were performed in triplicate. (B)–(C)–(D) Densitometric analysis was performed with Scion Image Software. Values are expressed by comparing the data obtained after EGF stimulation with those obtained without EGF (B); by comparing the data obtained after transfection with NEU3 with those obtained after transfection with the empty vector (mock) (C); by comparing the data obtained after gefitinib treatment with those obtained without gefitinib administration (D). Statistical analyses were performed using Student’s t-test. Values are presented as means ± standard error (SE). *p<0.05, **p<0.01, and ***p<0.001 (Student’s t-test).

In Fig 7, Akt phosphorylation levels in the different conditions are reported. In healthy HSAEC1 cells, EGF stimulated Akt phosphorylation (2-fold), only in the absence of NEU3 overexpression and without gefitinib treatment (Fig 7A and 7B). In HCC4006 and H1734 cell lines we observed a significant Akt activation after EGF stimulation in all conditions, except when cells were treated with gefitinib; Akt activation was restored to a certain extent, by the overexpression of sialidase NEU3 (Fig 7A and 7B).

As shown in Fig 7C, NEU3 overexpression was confirmed to lead to an increase in Akt phosphorylation in the healthy untreated lung cells. On the other hand, a significant effect of NEU3 on Akt phosphorylation was also detected in HCC4006 and H1734 cell lines, leading to a 3- and 2-fold increase, respectively, when the cells were treated with gefitinib and stimulated with EGF.

Gefitinib administration did not affect Akt phosphorylation level in healthy HSAEC1 lung cells, (Fig 7A and 7D). Conversely we observed a strong inhibition, mediated by gefitinib, of Akt activation in both HCC4006 and H1734 cell lines (Fig 7D).

## Discussion

The deregulation of sialidases drives the development of cancer. In particular, NEU3 is up-regulated in most tumours, including NSCLC, where about one third of cases displays NEU3 overexpression at various levels (at either RNA or protein status)[28]. Furthermore, EGFR regulation by NEU3 is a recent discovery. Our recent finding that sialidase NEU3 actively promotes EGFR autophosphorylation and is responsible for the increased viability of colorectal cancer cells [21] prompted us to investigate the effect of this sialidase overexpression in NSCLC. In fact, in this type of cancer EGFR is considered one of the most potent driver in tumour development in about 15–20% of cases and for which targeted therapies have been developed and introduced with success into the clinical setting. This was done by comparing the effect of EGF administration, *NEU3* overexpression and treatment with a specific EGFR-targeted therapy, gefitinib, in a healthy lung mucosa cell line (HSAEC1) and in two representative NSCLC cell lines, one of which sensitive (HCC4006, carrying a classical deletion in EGFR exon 19) and one resistant (H1734, with a EGFR wild-type gene status and carrying a KRAS exon 2 mutation) to gefitinib. Comparing data obtained through MTT test and PI staining, we could distinguish between two possible effect of gefitinib: inhibition of cell proliferation and cell death. Our results showed that, although treatment with increasing gefitinib concentrations lead to increasing cell death, the predominant effect of gefitinib administration on healthy and sensitive cells is the inhibition of cell proliferation. This was not unexpected in consideration of the fact that EGFR, the target of this TKI inhibitor, is mainly involved in cell proliferation pathways instead of cell death pathways.

We subsequently evaluated the level of NEU3 expression in the two NSCLC cell lines in comparison to the quantity observed in the normal mucosa cell line and we found that none of the two NSCLC cells displayed intrinsic *NEU3* overexpression, thus rendering these two models suitable to the perturbation of *NEU3* expression through transfection with a plasmid able to lead to a constitutively overexpression of such a sialidase.

Upon transfection with NEU3, Q-PCR experiments showed an increase in NEU3 mRNA levels of 50-100-30-fold in HSAEC1, HCC4006 and H1734 cell lines, respectively; moreover, an increase of 2 folds in protein expression, respect to endogenous NEU3 expressed by mock cells, was detected through Western-blot in all cell lines. Subcellular fractionation also showed that NEU3 is found in the membrane fraction, identified by the presence of transferrin receptor. However, we did not observe any variation in cell viability, in comparison to the control and irrespective of the administration of gefitinib, both at a concentration of 1 μM and at a concentration of 27 nM, corresponding to HCC4006 cells IC_50_. A slight increase in cell viability was found only in the *KRAS* mutant cell line in the absence of gefitinib, this value subsequently decreasing when the anti-EGFR drug is administered. However, these minor variations are not statistically significant. Our results are in contrast with those observed in colorectal cancer cells, where a significant increase in cell viability was observed in an EGFR-hyper-stimulated cell line upon *NEU3* overexpression [21]. However, we should remind the differences between adenocarcinomas of the colon-rectum and of the lung as regard the activation of EGFR. EGFR is mainly stimulated through protein overexpression in colorectal adenocarcinoma, whereas in adenocarcinoma of the lung it is mainly activated through hyper-activating point mutations occurring in its tyrosine kinase domain. In fact, EGFR was found to be downregulated in all the cell lines analysed in this work. Therefore, our data seems to indicate that NEU3 can facilitate the interaction of EGFR monomers in the presence of EGFR overexpression, but not when EGFR is normally expressed, or even downregulated, but activated by alterations of its intrinsic structure (occurrence of point mutations).

The analysis of EGFR phosphorylation explains the data of cell viability. Upon EGF administration, in the normal (HSAEC1) and in the *KRAS* mutant (H1734) cell lines we observed a significant stimulation of EGFR phosphorylation, irrespective of *NEU3* overexpression, while no stimulation was observed in the *EGFR*-mutant cell lines, probably because EGFR is already hyper-activated at its maximum degree due to the presence of the hyper-activating mutation in the TKI domain. Therefore, there is no additive effect of EGF to EGFR mutation. After gefitinib treatment, the effect of EGF is abolished in the normal and in the *KRAS* mutant cell line, while, as expected, although not furtherly stimulated by EGF, EGFR phosphorylation was greatly diminished in HCC4006. These data confirm the absence of NEU3 effect on EGFR activation in NSCLC cells: the effect we have observed is only due to EGF stimulation. This finding is, again, in contrast with what we observed in colorectal cancer cell lines, where, although carrying a strong EGFR activation, the cells showed a further increase of EGFR phosphorylation upon NEU3 transfection [21].

The analysis of the two main EGFR downstream pathways, namely the MAP kinase axis and the PI3K-Akt axis, revealed an interesting and sometimes more complex situation. ERK phosphorylation is enhanced upon EGF stimulation and abolished upon gefitinib administration only in HSAEC1 and in HCC4006 cell lines, not in the H1734 cell line. This finding can be ascribed to the presence of the *KRAS* mutation in the last cell line, a feature which leads to a constitutive ERK activation even upon TKI treatment (indeed, H1734 cells are gefitinib resistant). However, following NEU3 transfection, a further increase of ERK phosphorylation can be observed in the normal cell line with respect to mock. This may suggest that NEU3 can activate the ERK pathway in an EGFR-independent way: the enzyme does not act through the EGFR pathway, because its overexpression has no effect on the levels of EGFR phosphorylation (Fig 5). However, after gefitinib treatment, the effect of NEU3 on ERK phosphorylation is completely abolished. This finding is not completely unexpected: in colon cancer cell lines, we found a great increase of ERK phosphorylation after NEU3 overexpression, whereas only a slight (but not null as in HSAEC1 cells) increase of EGFR phosphorylation was observed. Therefore, we can postulate that usually NEU3 acts weakly on EGFR but strongly on ERK phosphorylation, and the analysis of a semi-quantitative method such as Western-blot, also after comparison with the level of EGFR protein expression, is not sufficient to precisely estimate little variations of EGFR phosphorylation. Although we do not know at present how sialidase NEU3 could act on ERK, it is worth mentioning that previous data from Sasaki and coworkers [29] showed that, in response to insulin, NEU3 undergoes tyrosine phosphorylation and subsequent association with Grb2 adaptor protein, through its SH2 domain. Grb2 thus becomes activated and can mediate ERK activation. This is well in accordance with a recent work [30] showing that NEU3 could be an integral membrane protein, interacting with different proteins on both sides of the membrane.

A more complex situation was observed for the PI3K-Akt pathway by the evaluation of the levels of Akt phosphorylation. As expected, Akt phosphorylation was observed after EGF administration and was repressed after gefitinib treatment. However, we found an effect of NEU3 on Akt activation: NEU3 overexpression lead to an increase of Akt phosphorylation in the healthy untreated cell line and to an attenuation of the reduction in pAKT caused by gefitinib in HCC4006 and H1734 cell lines. These results may indicate that NEU3 is also able to act indirectly on Akt, since gefitinib, an EGFR-specific drug, is not able to fully abolish the NEU3-mediated Akt phosphorylation. Therefore, we can conclude that NEU3 acts in different ways on the EGFR-downstream pathways: i) directly through EGFR on the ERK pathway, ii) both directly and indirectly with respect to EGFR on the Akt pathway. The last finding may also suggest that NSCLCs may benefit from a combinatorial effect of EGFR and Akt blockade when NEU3 is overexpressed.

Finally, a corollary of our results comes from the analysis of the behaviour of the HSAEC1 normal cell line under gefitinib administration. Unexpectedly, in this condition cell viability experiments showed that, although no EGFR gene mutations are present, the cells are slightly sensitive to the EGFR TKI (significantly less—about 18-fold–with respect to the EGFR-mutant cell line). These results suggest that EGFR wild-type cases may benefit from EGFR-targeted therapies directed against the EGFR TKI domain. At molecular level, a weak EGFR phosphorylation, as well as ERK and Akt phosphorylation were observed only in the HSAEC1 cell line but not in the H1734 cell line, that is *EGFR* wild-type but *KRAS* mutated. Gefitinib significantly decreased, but not abolished, all the activations, especially ERK phosphorylation which was at a lower level with respect to the other phosphorylations. NEU3 overexpression led to an increase of the activation of EGFR downstream pathway. Our results are not completely unexpected: randomized clinical trials have demonstrated that a subgroup of EGFR wild-type patients may have a benefit (significantly lower with respect to EGFR mutated patients) from TKI administration [31,32]. On this topic, a recent contribution retrospectively investigating stage IV EGFR wild-type patients who relapsed on first-line chemotherapy and who were treated with TKI or with chemotherapy, revealed that in general the EGFR TKI treatment was inferior as compared to chemotherapy, but in never-smoker patients the two treatments were comparable [33]. On the other hand, although some guidelines suggest that EGFR TKI can be considered for the management of cases with *EGFR* wild-type gene sequence, the role of EGFR-targeted therapies in this subgroup of patients is far from understood. Our results, especially those obtained in the presence of a forced NEU3 overexpression, may indicate that EGFR wild-type cases, carrying NEU3 overexpression, may represent the group of patients who can profit at most from EGFR TKI therapies although carrying a normal EGFR gene status, thus theoretically opening to a diagnostic role for NEU3 expression.

In conclusion, our results integrate the data obtained in colorectal cancer and enlarge the knowledge about the interplay between EGFR and NEU3: the main effect of NEU3 is to facilitate of the interaction of EGFR monomers, leading to the activation of EGFR-downstream pathways in the absence of EGFR activating point mutations. The ERK pathway represents the first axis of activation (since it is completely abolished after EGFR TKI administration); on the Akt pathway different activations (direct and indirect, since gefitinib decreases but not fully abolishes its activation) can be observed. Finally, our results, which must be confirmed also on patients’ cohorts, may indicate that patients with high NEU3 expression levels may benefit from EGFR TKI or by combinatorial treatments with Akt inhibitors.
